# Comparative morphometric analysis of the clavicle in male and female human cadavers: Implications for forensic identification

**DOI:** 10.6026/973206300220465

**Published:** 2026-01-31

**Authors:** Mayuri V Ghorpade, Deepali P Onkar, Manjusha K Tabhane, Sanika S Matey

**Affiliations:** 1 Department of Anatomy, N K P Salve Institute of Medical Sciences, Nagpur, Maharashtra, India

**Keywords:** Forensic anthropology, clavicle, sexual dimorphism, morphometry, sex determination, skeletal identification

## Abstract

Forensic sex estimation from fragmented skeletons challenges anthropology when pelvis and skull are absent. This study analyzed 120 dry human clavicles (60 male, 60 female) using maximum length, mid-shaft circumference and sternal end breadth and acromial end breadth measurements. All parameters demonstrated significant sexual dimorphism (p<0.001), with male clavicles consistently larger and discriminant analysis achieving 88.3% classification accuracy. Mid-shaft circumference and maximum length provided the strongest predictive values. Clavicle morphometrics advance forensic anthropology by offering reliable sex determination from isolated upper limb bones.

## Background:

Forensic anthropology is of paramount importance to the identification of human remains, especially in medico-legal cases that involve decomposition, mutilation or skeletonization. The creation of a biological profile (age, sex, stature and ancestry) is the main step in the identification process. Of these variables, the determination of sex has been widely regarded as the most fundamental, as it has the advantage of effectively reducing by one-half the possible pool of missing persons and is a prerequisite to the correct estimation of age and stature [1]. While the pelvis and skull are traditionally considered to be the most sexually dimorphic elements of the human skeleton, they are often recovered in a fragmented or incomplete state as a result of taphonomic processes [2]. There is therefore a great need to develop reliable standards for sex estimation based on postcranial bones that are resistant to environmental degradation. The clavicle or the collarbone is a long bone with a high survival rate in forensic applications because of the compact structure and relative density. It is the first bone to become ossified in the fetus and the last to complete epiphyseal union, which makes it unique in the development of the human [3]. Anatomically the clavicle is a strut that connects the scapula and sternum, which carries the force from the upper extremity to the axial skeleton. Due to the difference in the magnitude of these forces between the sexes because of the difference in muscle mass and occupational physical activity, the clavicle has significant sexually dimorphic characteristics [4]. Historical work has proposed that the clavicle is a good sex indicator; accuracy rates are comparable to that of the long bones in the limbs. Early morphometric studies established the fact that male clavicles tend to be longer, thicker and more curved than females [5]. However, skeletal metrics are known to be population specific; standards based on a single geographic or ancestral group often have little or no applicability when applied to a different group due to genetic, nutritional and environmental differences [6]. For example, discriminant functions created for American populations may not be applicable to Asian or European populations without large error. Despite the potential of the clavicle, there is a lack of wide-ranging studies, combining several linear dimensions with a strong statistical modelling of modern populations. Furthermore, many of the current studies are based on osteological collections made early in the 20th century and may not be representative of the secular trends in body size in modern populations [7]. Recent literature therefore highlights the need to update forensic databases to take into consideration these secular changes in order to maintain the accuracy of the identification protocols [8]. Therefore, it is of interest to fill in this gap by performing a detailed morphometric analysis of the clavicle in a controlled sample of cadaveric clavicle.

## Materials and Methods:

## Study design and setting:

This cross-sectional, observational study was conducted in the Department of Anatomy and Forensic Medicine at a tertiary academic medical center. The duration of the study was eighteen months.

## Sample size and selection:

The sample consisted of 120 adult human clavicles (60 male and 60 female) obtained from cadavers during routine dissection and autopsy procedures. The age of the subjects ranged from 25 to 65 years.

## Preparation of bones:

The clavicles were removed and put through a standardised maceration process. Soft tissues were removed by dissection and then the tissue was boiled in water with a mild detergent solution. The bones were then dried in the air at room temperature for 72 hours. All the specimens were labeled with a unique identification number to blind the observer regarding the sex of the bone during the measurement phase.

## Morphometric measurements:

Measurements were made with the help of digital Vernier caliper (precision 0.01 mm) and osteometric board. All measurements were measured in millimeters. To reduce inter-observer error, all of the measurements were made by one primary investigator. Intra-observer error was determined by re-measuring 20 randomly selected bones 2 weeks after the first data collection.

The following parameters were tested:

[1] Maximum Length (ML): Maximum length from the sternal to the acromial end of the clavicle (a measuring instrument known as the osteometric board is used).

[2] Mid-shaft Circumference (MSC): The circumference at the midpoint of the shaft measured utilizing non-elastic measuring tape checked with the caliper.

[3] Sternal End Breadth (SEB): Maximum anteroposterior diameter of the articular surface of sternal end.

[4] Acromial End Breadth (AEB): The greatest anterior-posterior diameter of the articular surface of the acromial end.

## Statistical analysis:

Data were entered into Microsoft Excel and analyzed with the use of Statistical Package for the Social Sciences (SPSS) version 26.0. Data Analysis: Descriptive statistics (Mean, Standard Deviation and Range) was calculated for all the variables, separating by sex. An Independent Student's t-test was used between the means for the male and female groups to determine the significance of the sexual dimorphism. P < 0.05 was considered as statistically significant. Demarking points (DP) were calculated to identify ranges that were exclusive to one sex. Discriminant Function Analysis (DFA) was done to obtain the coefficients for sex classification and to find the percentage of accuracy of each variable taken individually and in combination.

## Results:

The study was based on 120 clavicles. Intra-observer error testing found there were no statistically significant differences between measure sessions, proving the reliability of the method of data collection. The results showed that while male clavicles were dimensionally larger than female clavicles, they were larger for all four measured parameters. Table 1 shows the descriptive statistics of four morphometric parameters. The average value of Maximum Length (ML) for males was 152.45 +- 6.82 mm, while that of the females was 139.12 +- 5.94 mm. The Mid-shaft Circumference (MSC), a good indicator of the degree of robusticity, revealed a mean of 39.22 +- 3.10 mm for males and 32.45 +- 2.85 mm for females. As shown in Table 1, the difference in the mean of male and female was found to be statistically significant for all the variables (p<0.001). The highest t-value for any trait was found for Mid-shaft Circumference (12.43); this trait appears to be the most sexually dimorphic trait measured, followed closely by Maximum Length. Results of the Discriminant Function Analysis are presented in Table 2. Univariate analysis was carried out to determine the accuracy of individual variables in predicting sex. Mid-shaft Circumference gave the most individual accuracy (86.7%), followed by Maximum Length (84.2%). The articular end measurements (SEB and AEB) had lower, but significant, accuracy rates. To maximize classification accuracy, a stepwise multivariate analysis was conducted combining the variables. The combination of Maximum Length and Mid-shaft Circumference yielded the best predictive model. Table 3 demonstrates that combining variables increases accuracy. Using all four variables resulted in 90.0% classification accuracy. However, the combination of just two variables (ML + MSC) achieved 88.3%, indicating that length and thickness are the primary drivers of dimorphism in the clavicle.

## Discussion:

The identification of sex from skeletal remains is one of the foundations of forensic anthropology. While the pelvis is still the gold standard, the results of this study confirm that the clavicle is a statistically robust alternative to the pelvis for sex determination. The results show that male clavicles are much larger than female clavicles in length, circumference and articular widths, in line with the general pattern of sexual dimorphism in the human skeleton which is due to genetic, hormonal and environmental factors. The results of this analysis showed that Mid-shaft Circumference (MSC) is the single most dimorphic parameter with an accuracy of 86.7% on its own. This finding is consistent with the mechanical loading hypothesis. Males generally have more upper body musculature than females. The clavicle is a site of attachment for some of the major muscles such as the pectoralis major, deltoid, trapezius and sternocleidomastoid muscle. Increased activity of muscles increases tensile and compressive loads on the bone and promotes periosteal apposition, leading to a thicker shaft [9]. This explains why circumference which expresses robusticity often is better than length in discriminatory power. The maximum length obtained (ML) was also found to have high accuracy (84.2%). The longer length in males is attributed to a longer time of growth. Males are generally found to have a delay in the onset of puberty than females with the result of having a prolonged period of epiphyseal growth before fusion occurs [10]. This physiological difference causes generally longer long bones in males. The results of this study are consistent with the established literature but provide refined population-specific information. Previous studies of samples from America found classification accuracies of between 85% and 92% using the clavicular measurements [11]. Similarly, the studies carried out on the Indian populations have reported an accuracy rate between 80 and 88% [12]. The overall accuracy of 90.0% obtained in this study by using multivariate analysis is at the higher end of this spectrum and this validates the methodology followed. However, there are variations in comparison with absolute dimensions. The mean male clavicle length in this study (152.45 mm) is slightly less than values reported for the African American populations (approx. 158 mm) but greater than those reported for some South Asian populations (approx. 147 mm) [13]. These discrepancies highlight the importance for specificity to the population for forensic anthropology. Secular trends, nutritional status and physical labor patterns play an important role in skeletal development [14]. Therefore, use of a discriminant function based on a Western population in an Asian or African sample could cause misidentification. The statistical analysis underlines the usefulness of the "Demarking Point" a concept introduced to define ranges where there is no overlap between the sexes. While there was some overlap in the distributions of male and female measurements in this study (as is evident in Table 1), the multivariate functions were good at separating the groups. "In mass disaster scenarios or cases in which remains are comingled the ability to take advantage of the clavicle, a bone which often survives fragmentation better than the flat bones of the pelvis, is invaluable [15]." Furthermore, the results in this study suggest that even if the ends of the clavicle are damaged (a common taphonomic occurrence), the Mid-shaft Circumference alone is a very reliable estimate of sex [16]. This is important for crime scene investigators and anthropologists who have to work with incomplete remains. While the results are good, this study has limitations. The size of the sample (N=120), although statistically large enough for this study, is small by comparison to large osteological databases. Additionally, the study hardly considered the handedness of the individuals or the occupation history that could affect the asymmetry of the sides and the robusticity [17]. Future research should focus on 3D geometric morphometrics to analyse the curvature of the clavicle, which may add another layer of discriminatory power [18].

## Conclusion:

We show that the clavicle is a highly sexually dimorphic bone that can be used for forensic sex determination. The male clavicle is statistically larger than the female clavicle in all dimensions measured. Mid-shaft circumference and maximum length were found to be the best predictors of sex with an overall combined accuracy approaching 90%. Thus, we show the application of clavicular measurements as one of the main methods of biological profiling in the absence or impairment of pelvic or cranial elements. The discriminant functions produced in this study give a valuable tool for forensic experts, which increase the chances of correct identification in medico-legal investigations.

## Figures and Tables

**Table 1:** Descriptive statistics and comparison of clavicular dimensions (in mm)

**Parameter**	**Sex**	**N**	**Mean**	**SD**	**Min**	**Max**	**t-value**	**p-value**
Max Length (ML)	Male	60	152.45	6.82	138.5	168.2	11.45	<0.001*
	Female	60	139.12	5.94	125.4	151		
Mid-shaft Circ (MSC)	Male	60	39.22	3.1	33.1	46.5	12.43	<0.001*
	Female	60	32.45	2.85	27	38.2		
Sternal End (SEB)	Male	60	22.85	2.15	18.5	27.1	8.92	<0.001*
	Female	60	19.42	1.98	15.2	23.8		
Acromial End (AEB)	Male	60	20.15	1.85	16.2	24.5	7.65	<0.001*
	Female	60	17.65	1.72	14	21.3		
Significant at
p < 0.001

**Table 2:** Accuracy of sex determination using univariate discriminant function analysis

**Variable**	**Sectioning Point**	**% Accuracy Males**	**% Accuracy Females**	**Overall Accuracy**
Maximum Length	145.78	83.30%	85.00%	84.20%
Mid-shaft Circ	35.83	86.70%	86.70%	86.70%
Sternal End Breadth	21.13	76.70%	78.30%	77.50%
Acromial End Breadth	18.9	73.30%	75.00%	74.20%

**Table 3:** Stepwise multivariate discriminant function analysis results

**Function Combination**	**Wilks' Lambda**	**Chi-Square**	**Canonical Correlation**	**Overall Accuracy (%)**
ML + MSC	0.412	104.23	0.765	88.30%
ML + MSC + SEB	0.405	106.15	0.771	89.20%
All 4 Variables	0.398	108.44	0.776	90.00%
